# Noncardiogenic Pulmonary Edema after Amlodipine Overdose without Refractory Hypotension and Bradycardia

**DOI:** 10.1155/2015/546012

**Published:** 2015-05-05

**Authors:** M. Hedaiaty, N. Eizadi-Mood, A. M. Sabzghabaee

**Affiliations:** Clinical Toxicology Department, Isfahan Clinical Toxicology Research Center, Noor Hospital, Isfahan University of Medical Sciences, Isfahan 81458-31451, Iran

## Abstract

Amlodipine overdose can be life-threatening when manifesting as noncardiogenic pulmonary edema. Treatment remains challenging. We describe a case of noncardiogenic pulmonary edema without refractory hypotension and bradycardia after ingestion of 500 milligram amlodipine with suicidal intent. Mechanical ventilation, dexamethasone, atrovent HFA (ipratropium), pulmicort inhalation, and antibiotic therapy were used for the management. Length of hospital stay was 11 days. The patient was discharged with full recovery.

## 1. Introduction

Amlodipine, a dihydropyridine group of calcium channel blockers (CCBs), constitutes the leading form of cardiovascular drug overdose and has been implicated in several deaths resulting from such overdose [1, 2]. It has half-life of 30–50 hours with a large volume of distribution (21 liter per kilograms) [1]. It has also a low metabolic clearance with the advantage of using a once-daily dosage [3].

Treating patients with amlodipine overdose can be challenging [4]. Patients severely poisoned can develop profound refractory bradycardia, hypotension, acute kidney injury, and either cardiogenic or noncardiogenic pulmonary edema [5].

Here we report a case of amlodipine overdose with noncardiogenic pulmonary edema without refractory hypotension and bradycardia which was managed supportively.

## 2. Case Report

A 36-year-old woman was admitted to our poisoning emergency department with recurrent vomiting and generalized muscular pain 11 hours after ingestion of 100 tablets of amlodipine five milligram. She had a suicidal intent. She had gone to a local health center three hours after the consumption. Gastric decontamination had been performed for her at that center. Then she had been discharged with her own consent.

On admission, she had a blood pressure of 95/60 mm Hg in the supine position with a pulse rate of 99 per minute and respiratory rate of 21 per minute (Table 1). She was afebrile, conscious, and anxious. Other cardiac and respiratory manifestations were normal. Pulse oximetry (SpO_2_) showed 93% on room air. She denied concomitant consumption of alcohol or any other drugs. Comprehensive toxicology analysis of urine was negative for opioids, morphine, alcohols, amphetamines, and so forth. There were no signs of head trauma or focal neurologic signs. She was hospitalized in an intensive care unit with respect to high toxic ingestion. Routine laboratory tests on admission were as follows: white blood cells (12.6 × 10^9^ per liter; normal range: 4–10 × 10^9^ per liter); 90% neutrophil; serum urea (BUN) 21 milligrams per deciliter (mg/dL); creatinine (Cr) 1.6 mg/dL; serum calcium 8.4 mg/dL; phosphorus 5.8 mg/dL; and glucose plasma level 184 mg/dL. Liver function tests, sedimentation rate (ESR), and serum electrolytes were within the normal limits. Venous blood gas analysis showed respiratory alkalosis (pH 7.54, carbon dioxide tension 18 millimeter of mercury, Bicarbonate 15.1 milimol per liter) (Table 2). Electrocardiography demonstrated sinus tachycardia with normal PR, QRS, and Q-T intervals. Chest X-ray performed immediately after admission was normal.

After four hours, blood pressure decreased to 85/50 millimeter of mercury (mm Hg). She received one-liter normal saline as bolus infusion. Hourly urine output was initially below 0.5 milliliter per kilograms (mL/kg) body weight but it improved after infusion of crystalloid intravenous (IV) fluids and vasopressors (dopamine hydrochloride drip at a rate of 5–10 micrograms per kilograms per minute) for ten hours. She had recovered from renal failure within 48 hours.

Next day she began to experience gradual respiratory distress including developed tachycardia, tachypnea (respiratory rates = 25) (Table 1), and mild agitation. Fine inspiratory crackles in both lungs at auscultation were present. Arterial blood gas (ABG) showed hypoxia arterial oxygen tension = 62 mm Hg and oxygen saturation of 85%. The FiO_2_/PaO_2_ ratio was less than 200. Central vein pressure was within normal range (14 centimeter of water). Chest X-ray revealed bilateral fluffy shadows in the lower zones of both lung fields without cardiomegaly. Echocardiography was performed and ejection fraction was 60%. No evidence of diastolic dysfunction was observed.

She was intubated under sedation with midazolam (0.1 milligram per kilograms) and ventilated (initial settings: synchronized intermittent mechanical ventilation (SIMV) mode, Fraction of Inspired Oxygen (FiO_2_) = 70%; tidal volume 6–8 mL/kg; positive end expiratory pressure (PEEP) = 5 cm H_2_O; respiratory rate (RR) = 10/minute; pressure support (PS) = 10 mm Hg). After 10 hours, she presented with acute respiratory distress syndrome (ARDS) and the setting was adjusted to: FiO_2_ = 40%, PEEP = 10 cm H_2_O, RR = ten per minute, and PS = 15 cm H_2_O. The patient received dexamethasone (8 mg, three times daily), ipratropium bromide inhalation aerosol (three milliliter vial, three times daily), and pulmicort inhalation (one milligram per day). The results regarding the vital signs, O_2_ saturation, VBG, BUN, and Cr during hospitalization in an intensive care unit have been reported in Tables 1 and 2. Chest X-ray on day three showed typical batwing appearance without cardiomegaly which was suggestive of ARDS.

After 11 days of hospitalization she was extubated and transferred to the ward. She underwent psychiatric evaluation and was discharged without any complications.

## 3. Discussion

There are no standardized guidelines for managing severe amlodipine intoxication because of limitation in the number of describing surveys [4, 6–10].

Gastrointestinal decontamination in amlodipine overdose is beneficial when used within the one hour of consumption [11]. Also activated charcoal had been effective when given during the first 24 hours after drug ingestion [12]. In our case gastrointestinal decontamination for three hours and activated charcoal for 11 hours after ingestion were started.

Different pharmacologic therapies available for amlodipine overdose with persistent hypotension or myocardial depression include inotropic support with adrenergic agents, glucagon, IV infusion of calcium, hyperinsulinemia-euglycemia therapy, and extracorporeal membrane oxygenation in refractory shock [4, 7, 10, 11, 13]. In this case, she had mild hypotension without cardiac conduction defects; therefore, she received only crystalloid and dopamine infusion and stabilized in a short time period.

Our patient developed noncardiogenic pulmonary edema on day three. Amlodipine is a dihydropyridine that selectively blocks L-type calcium channels in smooth muscle and myocardial depressant activity at toxic levels so patients may present with cardiogenic pulmonary edema [13, 14]. Some studies also reported cases with catastrophic shock and noncardiogenic pulmonary edema [9, 10, 15]. The mechanism of noncardiogenic pulmonary edema in patients with CCB overdose is not well known. Excessive pulmonary capillary transudation due to selective precapillary vasodilatation causes an increase in transcapillary hydrostatic pressure and ultimately interstitial edema [16, 17].

In our case severe hypotension was not observed, so interstitial edema may be caused by other ways such as blocking other types of calcium channels, the cytochrome P450 isoenzyme system effects [18, 19], or P-glycoprotein-mediated transport [20]. Also cardiac ejection fraction was 60%; therefore, fluid overload could not be reason of pulmonary edema. Considering age, sex, serum urea, creatinine level, and glomerular filtration rate, she suffered from mild and transient kidney injury. So kidney injury could not be reason of pulmonary edema.

Severe CCB poisoning is often associated with significant hyperglycemia due to L-type calcium channel in pancreatic *β*-cells [21], as well as dysregulation of the insulin-dependent or phosphatidylinositol three-kinase pathway [22]. However, in this case glucose plasma levels were between 151 and 184 mg/dL for initial four days that may show a role in the ultimate degree of toxicity.

Another finding in this case was the development of mild respiratory and metabolic alkalosis. However, metabolic acidosis has been reported in most of the reported cases [5–16]. Metabolic acidosis could be resulting from systemic hypotension and acute kidney injury.

ARDS manifests with diffuse alveolar inflammation and increased pulmonary vascular permeability resulting in hypoxemia [23]. A survey has shown mortality benefits with lower tidal volume of 6 mL/kg, keeping plateau pressure below 30 cm H_2_O and PEEP adjusted to optimize alveolar recruitment without causing overdistention [24]. Our patient developed ARDS related to noncardiogenic pulmonary edema. She was treated with mechanical ventilation, dexamethasone, ipratropium bromide inhalation aerosol, and pulmicort inhalation which improved outcome and successful liberation from ventilator around 11 days.

Although high-dose insulin and extracorporeal life support were the interventions supported for the patients with severe CCB [25], supportive management might be useful in the treatment of noncardiogenic pulmonary edema after amlodipine overdose without refractory hypotension and bradycardia.

## Figures and Tables

**Table 1:** Vital signs (systolic and diastolic blood pressure, respiratory rates, and pulse rate) of patient during hospitalization (on admission and at 8.00 a.m. each day).

Time of hospitalization (day)	Systolic blood pressure (mmHg)	Diastolic blood pressure (mmHg)	Pulse rate (per min)	Respiratory rates (per min)
On admission	95	60	99	21
1	90	50	108	21
2	90	54	104	v
3	105	55	100	v
4	101	59	75	v
5	115	56	104	v
6	105	55	96	v
7	116	67	85	v
8	98	59	88	v
9	113	75	80	v
10	112	78	91	v
11	103	60	61	v
12	115	75	73	18

min:  minutes; mmHg: millimeters of mercury;  v: treatment with  mechanical ventilation.

**Table 2:** Different variables of the patient (on admission and at 8.00 a.m. each day): intake and output volume, blood urea nitrogen (normal value ranges: 9–20 mg/dL), creatinine (normal value ranges: 0.7–1.4 mg/dL), peripheral venous blood gases including partial pressure of carbon dioxide (normal value ranges: 40–52 mmHg), venous blood pH (normal value ranges: 7.31–7.41), bicarbonate (normal value ranges: 22–27 mEq/L), and saturation of peripheral oxygen (normal present ranges: 90–100%).

Time of hospitalization (day)	Volume intake (mL)	Volume output (mL)	Blood urea nitrogen (mg/dL)	Creatinine (mg/dL)	PCO_2_ (mmHg)	PH	HCO_3_ (mEq L)	SpO_2_%
On admission	—	—	21	1.6	18	7.54	15.1	93
1	2250	700	24	1.3	18	7.54	15	84
2	3950	250	37	2.3	26	7.24	15.2	85
3	3100	1000	45	1.4	24	7.44	15.9	89
4	4850	1200	32	1.2	27	7.46	18.6	98
5	3650	2300	27	0.8	29	7.49	21.4	94
6	3400	2100	18	0.6	29	7.49	38	96
7	3850	5400	12	0.6	37	7.50	28.3	99
8	3850	8150	14	0.6	51	7.45	34.6	100
9	4300	3700	14	0.8	41	7.50	31.2	96
10	3900	5800		—	42	7.40	31.3	95
11	3100	2950	8	0.8	42	7.49	31.3	97
12	3100	2900			—	—		

mg/dL: milligram per deciliter; mEq/L: milliequivalents per liter; mmHg: millimeters of mercury; mL: milliliter; PCO_2_: partial pressure of carbon dioxide; HCO_3_: bicarbonate; SpO_2_: saturation of peripheral oxygen.
